# A two-stage search strategy for detecting multiple loci associated with rheumatoid arthritis

**DOI:** 10.1186/1753-6561-3-s7-s72

**Published:** 2009-12-15

**Authors:** Pritam Chanda, Aidong Zhang, Lara Sucheston, Murali Ramanathan

**Affiliations:** 1 Departments of Computer Science and Engineering, State University of New York, Buffalo, New York 14260, USA; 2 Department of Biostatistics, State University of New York, Buffalo, New York 14260, USA; 3 Department of Pharmaceutical Sciences, State University of New York, Buffalo, New York 14260, USA

## Abstract

Gene × gene interactions play important roles in the etiology of complex multi-factorial diseases like rheumatoid arthritis (RA). In this paper, we describe our use of a two-stage search strategy consisting of information theoretic methods and logistic regression to detect gene × gene interactions associated with RA using the data in Problem 1 of Genetic Analysis Workshop 16. Our method detected interactions of several SNPs (single-SNP and SNP × SNP) that are located on chromosomal regions linked to RA and related diseases in previous studies.

## Background

The risk of developing many common and complex diseases such as cancer and autoimmune disease involve complex interactions between multiple genes and several endogenous and exogenous environmental factors (or covariates). Rheumatoid arthritis (RA) is a complex genetic disease in which it is hypothesized that several loci contribute to disease susceptibility. Information theoretic methods are among the most promising approaches for genetic association studies and have been used for genetic analysis [1,2] and analysis of gene × gene interactions [3,4]. In this paper, we describe our use of a two-stage strategy consisting of an information theoretic search followed by logistic regression to detect gene × gene interactions associated with RA using selected genomic regions from the genome-wide scan data from the North American Rheumatoid Arthritis Consortium, which comprises 868 cases and 1194 controls. Data were provided as Problem 1 of Genetic Analysis Workshop 16.

## Methods

### Interaction information as measure of association

Let *X*_*i *_denotes a genetic random variable representing the genotypes at locus *L*_*i*_. We assume *L*_*i *_is biallelic (with alleles *A *and *a*) with three possible genotypes (*AA*, *Aa*, and *aa*). The uncertainty of *X*_*i *_is given by Shannon's entropy [5] as

Given a set of such genetic variables *S *= {*X*_1_; *X*_2_;...; *X*_*k*_}, the interaction information among the *k *variables (referred to as *k*-way interaction information or *KWII*) is defined as the amount of information (redundancy or synergy) present in the set of variables that is not present in any subset of these variables [4]. For the variables in set *S*, the *KWII *can be written succinctly as an alternating sum over entropies (*H*) of all possible subsets *τ *of *S *using the difference operator [6]:

Let *C *be the random variable representing the disease status (phenotype variable) of *RA*. Then *KWII*(*S*;*C*) = *KWII*(*X*_1_; *X*_2_;...; *X*_*k*_;*C*) is a measure of the association of the set of genetic variables in set *S *towards the disease phenotype variable *C *(i.e., how well the set explains the disease phenotype). The value of *KWII*(*S*;*C*) can be both positive and negative. We shall use only positive *KWII *values as the measure of association because larger positive values indicate stronger interaction (hence, higher association).

### Redundancy between combinations of variables

Let *S*_1 _= {*X*_1_; ...; *X*_*m*_} and *S*_2 _= {*Y*_1_; ...; *Y*_*m*_} be two sets (or combinations) of variables. Then the redundancy between *S*_1 _and *S*_2 _is given by the maximized average of pairwise linkage disequilibrium (LD) (*r*^2^) between variables from *S*_1 _and *S*_2_:

Such redundancies can arise because of LD between the variables across each set. For example, for a disease *C *that is caused by interactions between two untyped SNPs *D*_1 _and *D*_2_, let four marker loci be designated *X*_1_, *X*_2_, *X*_3_, and *X*_4 _such that *X*_1 _and *X*_3 _are in strong LD with *D*_1_, while *X*_2 _and *X*_4 _are in strong LD with *D*_2_. Then the *KWII*(*X*_1_;*X*_2_;C) and *KWII*(*X*_3_;*X*_4_;C) measure the association of the sets {*X*_1_;*X*_2_} and {*X*_3_;*X*_4_} for *C*, respectively. The redundancy between the combinations {*X*_1_;*X*_2_} and {*X*_3_;*X*_4_} is given by say, 0.5*(*r*^2^(*X*_1_, *X*_3_)+*r*^2^(*X*_2_, *X*_4_)) and existence of strong LD between *X*_1 _and *X*_3 _and between *X*_2 _and *X*_4 _will result in similar measures of *KWII *association for both sets, making one of the sets statistically redundant.

### Stage I: Single-nucleotide polymorphism (SNP)-combination search strategy

Let *S *be the set of all genetic (SNPs) and environmental (non-genetic) variables (e.g., sex) and *C *be the variable denoting the disease phenotype. The information theoretic metric *KWII*(*X*_1_;...;*X*_*k*_;*C*) is a measure of the association of the set of variables with the disease phenotype variable *C *(i.e., how well they explain the disease phenotype). Using this metric and a redundancy measure, we iteratively search for combinations of variables up to a fixed number (say *τ*) of iterations. Let the number of variables (except *C*) in a combination be defined as the "order" of the combination. In our method, we limit our search to up to second-order (or two-variable) combinations (i.e., we consider only {*X*_*i*_;*C*} and {*X*_*i*_;*X*_*j*_;*C*} combinations). Let *θ *be the set of variables and *ξ *be the set of associated combinations output by our search method. Initially, both *θ *and *ξ *are empty. In iteration = 1, the variable *X*_*k *_having highest *KWII*(*X*_*k*_;*C*) is selected; thus *θ *= {*X*_*k*_} and *ξ *= {(*X*_*k*_;*C*)}. Also *X*_*k *_is removed from *S*. In a subsequent iteration = *i *(*i*> 1), a new variable *X*_*j *_∈ *S *is considered for selection and its single variable and two-variable combinations are formed and *KWII *computed (using, Eq. (2)) with variables already selected in the previous iterations. At the same time each of the combinations formed are checked for redundancy with combinations already in *ξ *and of same order (using Eq. (3) and redundancy exceeding a threshold of 0.7). For example in iteration = 2, for *X*_*j *_∈ *S*, the combinations {*X*_*j*_;*C*} and {*X*_*k*_;*X*_*j*_;*C*} are formed and {*X*_*j*_;*C*} is checked for redundancy with {*X*_*k*_;*C*}. From all the new variables, the variable that has maximum *KWII *and all non-redundant combinations is selected. A variable with a redundant combination is dropped from consideration (i.e., removed from *S*) in subsequent iterations. Given the computational burden of determining redundancy with combinations of variables already selected, our selection procedure stops after a maximum of *τ *= 50 iterations. Thus, up to 50 variables with non-redundant combinations and highest *KWII *are selected. This stage yields a number of single and two-variable combinations and their *KWII *values, which are input to the second stage.

### Stage II

We conduct logistic regression analysis on the one- and two-variable combinations obtained by our information theoretic search using the methods outlined by Cordell [7] and the significance of each combination is determined. The full single-locus model is

where *r *is the probability of each individual being a case, *μ *corresponds to the mean effect, the terms *a *and *d *correspond to the additive and dominance coefficient effects of the tested SNP variable, *x *and *z *are dummy variables with *x *= 1, *z *= -0.5 for one homozygote genotype (*AA*), *x *= 0, *z *= 0.5 for the heterozygote genotypes (*Aa*), and *x *= -1, *z *= -0.5 for the other homozygote type (*aa*). The chi-square is used to compare the full single-locus model with the null model given by 0 values for both *a *and *d*.

For SNP × SNP interactions, fully saturated model following Cordell's notation [7] is

where *r *and *μ *are same as in Eq. (3), the terms *a*_1_, *d*_1_, *a*_2_, and *d*_2 _are the dominance and additive effect coefficients of the two SNPs, *i*_*aa*_, *i*_*ad*_, *i*_*da*_, and *i*_*dd *_represent their interaction coefficients, and *x*_*i *_and *z*_*i *_are dummy variables with *x*_*i *_= 1, *z*_*i *_= -0.5 for one homozygous genotype (*AA *or *BB*), *x*_*i *_= 0, *z*_*i *_= 0.5 for the heterozygous genotypes (*Aa *or *Bb*), and *x*_*i *_= -1, *z*_*i *_= -0.5 for the other homozygous genotype (*aa *or *bb*). An interaction is tested by the deviance of the full two-locus model from the model minus the interaction terms with chi-square test.

### Data

We have followed a candidate-gene-based approach and selected SNPs belonging to the candidate genes/regions in Table 1 for exploring both gene × RA and gene × gene × RA interactions using our two-stage approach. The start and end base-pair positions of each gene are obtained from http://www.pharmgkb.org/. Using the genes/regions from Table 1, we created the following three data sets for analysis:

**Table 1:** Candidate genes, associated genes/regions and number of SNPs (#s) in each

Gene	Chr	No. SNPs
*TNFRSF1B*	1	17
*PADI4*	1	8
*PTPN22*	1	10
*FCRL3*	1	10
*FCGR3A*	1	3
*FCGR3B*	1	4
*IL10*	1	6
*IL1A*	2	3
*IL1B*	2	9
*ITGAV*	2	27
*STAT4*	2	2
*CTLA4*	2	5
*BTLA*	3	2
*IL3*	5	2
*SLC22A4*	5	15
*IL13*	5	4
*IL4*	5	5
*HAVCR1*	5	11
*6p21.3*	6	1702
*MICA*	6	230
*HLA-C*	6	20
*NFKBIL1*	6	11
*LTA*	6	6
*TNF*	6	5
*HLA-DR*	6	21
*VEGFA*	6	6
*6q23*	6	1841
*OLIG3*	6	2
*TNFAIP3*	6	5
*IL6*	7	2
*IRF5*	7	4
*C5*	9	8
*DLG5*	10	20
*MS4A1*	11	12
*MHC2TA*	16	7
*CARD15*	16	10
*RUNX1*	21	3044
*MIF*	22	12

• 7087 SNPs selected for analysis using all genes/regions (Data Set 1)

• 5385 SNPs selected using all genes/regions except those that belong to only 6p21.3 and not to any other gene (Data Set 2)

• 3263 SNPs selected using genes not on chromosome 6 (Data Set 3)

Additionally, sex of the subjects and RA status were present in each data set as the environmental variable and the phenotype variable (*C*).

## Results

We have obtained many single-variable and two-variable interactions with the disease phenotype, only the combinations with high values of *KWII *are presented in Tables 2, 3, 4. The SNPs shown to be in genomic regions 6p21.3 and 6q23 do not overlap with any other gene. We found no interaction between the covariates sex and RA. Table 2 shows the single-variable combinations with *KWII *values greater than or equal to 95^th ^percentile of all the single-variable *KWII *obtained using our method for the respective data sets. Tables 3 and 4 show the two-variable combinations with *KWII *values greater than or equal to 95^th ^percentile of all the two variable *KWII *obtained using our method for the respective data sets. The 95^th ^percentile value for each data set is reported with each table where  denote the 95^th ^percentile *KWII *for combinations of order *i *and data set *j*. Additionally, to assess the overall strength of the *KWII *values we have obtained, we have calculated the *KWII *values of each single-variable combination for all 7088 variables, and 50,000 two-variable combinations randomly chosen from the list of 25,116,328 pairs of variables. The 95^th ^percentile of these were found to be  = 0.01 (one-variable combinations) and  = 0.004 (two-variable combinations). All interactions reported in Tables 2, 3, 4 have *KWII *higher than these values. We have detected several one-variable associations in 6p21.3, *HLA-DR*, and *RUNX1 *(Table 2) and also in 6q23 and *HLA-C *(with  <*KWII*<95^th ^percentile for respective data sets, not shown in tables). The strongest of the two-variable interactions are from the SNPs in region 6p21.3 (Table 3) obtained using Data Set 1. We have created the other two data sets because it was felt that several relatively weaker interactions are difficult to detect in the presence of the strongest interactions in 6p21.3. Using Data Sets 2 and 3, we found several two-variable *KWII *in genes *MICA *and *RUNX1 *(Table 3). Also several two-variable interactions are detected among SNPs in *HLA-C*, *HLA-DR*, *MICA*, and 6p21.3 (with  <*KWII*<95^th ^percentile for respective data sets, not shown in tables). Also we observed an interaction between rs11811771 (*PTPN22*) with rs2828104 (*RUNX1*) with *p*-value 2.7 × 10^-5 ^and *KWII *= 0.095. Separately we also calculated *KWII *for two-SNP combinations for Data Set 1 wherein the SNPs belong to different genes/genomic regions (Table 4).

**Table 2:** {*SNP*;C} interactions with KWII values ≥ 95^th ^percentile of the 1-variable KWII obtained for Data Set 1, Data Set 2, Data Set 3

SNP	Gene/genome region	KWII	*p*-Value^b^
Data Set 1: = 0.015^a^			
rs2395175	*HLA-DR*	0.195	0
rs660895	6p21.3	0.189	0
rs6910071	6p21.3	0.163	0
rs3763312	6p21.3	0.151	0
Data Set 2: = 0.075			
rs2395175	*HLA-DR*	0.195	0
rs7192	*HLA-DR*	0.094	0
rs3129871	*HLA-DR*	0.079	0
rs3129882	*HLA-DR*	0.075	0
Data Set 3: = 0.02			
rs731059	*RUNX1*	0.048	0
rs475142	*RUNX1*	0.024	1.3 × 10^-11^

^a^ denotes the 95^th ^percentile *KWII *for combinations of order *i *and Data Set *j*.

^b ^*p*-Value obtained using logistic regression

**Table 3:** {*SNP*_1_;*SNP*_2_;C} interaction with KWII values ≥ 95^th ^percentile of the two variable KWII obtained for Data Set 1 and consisting of SNPs only in 6p21.3 (and not in any candidate gene), for Data Set 2, and for Data Set 3)

SNP1-SNP2	Gene/genome region 1 - Gene/genome region 2	*KWII*	*p*-Value^b^
Data Set 1: = 0.07^a^			
rs2647050-rs2858332	6p21.3-6p21.3	0.144	0
rs9357152-rs2858332	6p21.3-6p21.3	0.098	0
rs9275141-rs2858331	6p21.3-6p21.3	0.092	0
rs7774434-rs2856718	6p21.3-6p21.3	0.08	0
rs9275371-rs7765379	6p21.3-6p21.3	0.076	3.35 × 10^-14^
rs660895-rs7755224	6p21.3-6p21.3	0.073	1.73 × 10^-7^
rs9357152-rs9275555	6p21.3-6p21.3	0.07	9.98 × 10^-8^
Data Set 2: = 0.02			
rs9263871-rs9263969	*MICA*-*MICA*	0.03	1.35 × 10^-8^
rs11967684-rs2523608	*MICA*-*MICA*	0.025	2.22 × 10^-14^
rs9263871-rs2596501	*MICA*-*MICA*	0.022	1.81 × 10^-5^
rs11967684-rs7755852	*MICA*-*MICA*	0.02	2.45 × 10^-8^
rs3873380-rs7755852	*MICA*-*MICA*	0.02	1.39 × 10^-9^
Data Set 3: = 0.01			
rs1542876-rs1513737	*RUNX1*-*RUNX1*	0.015	1.85 × 10^-6^

^a^ denotes the 95^th ^percentile *KWII *for combinations of order *i *and Data Set *j*.

^b ^*p*-Value obtained using logistic regression.

**Table 4:** The two-variable interactions with KWII values ≥ 95^th ^percentile (0.01) obtained using SNPs on gene × gene pairs on Data Set 1

SNP1-SNP2	Gene/genome region 1 - Gene/genome region 2	*KWII*	*p*-Value^a^
rs9275596-rs1542876	6p21.3-*RUNX1*	0.01914	2.05 × 10^-6^
rs2856725-rs1542876	6p21.3-*RUNX1*	0.01743	3.61 × 10^-5^
rs9275596-rs1041778	6p21.3-*RUNX1*	0.01471	8.25 × 10^-7^
rs7770216-rs563495	6p21.3-6q23	0.01444	4.15 × 10^-7^
rs7755852-rs2745443	6p21.3-6q23	0.01275	6.22 × 10^-7^
rs9275698-rs1883468	6p21.3-6q23	0.01262	4.51 × 10^-7^
rs4673260-rs12190331	*CTLA4*-6q23	0.01385	7.86 × 10^-8^
rs1206684-rs651084	6q23-*RUNX1*	0.01261	6.12 × 10^-5^
rs2844729-rs16984549	6p21.3-*RUNX1*	0.01255	5.71 × 10^-7^
rs2856725-rs1041778	6p21.3-*RUNX1*	0.01247	6.54 × 10^-6^

^a ^*p*-Value obtained using logistic regression.

**Figure 1:**
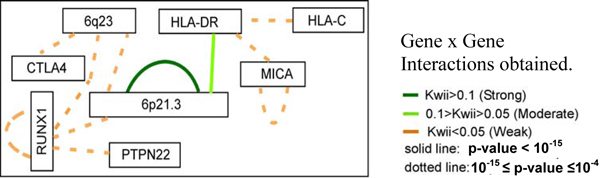
**Gene × gene interactions obtained**.

The *KWII *values of two-variable combinations greater than  = 0.004 are used to construct the gene × gene interaction diagram (Figure 1). We have categorized these interactions as: 1) strong (*KWII *≥ 0.1) in green, 2) moderate (0.1 > *KWII *≥ 0.05 in light green, and 3) weak in orange. Also, bold lines indicate *p*-values < 10^-15 ^while dotted lines denote 10^-15 ^≤ *p*-value ≤ 10^-4^.

## Discussion

We have used a two-stage strategy to search for single SNPs and SNP × SNP interactions associated with RA. Using our analysis on the candidate genes, we have found several strong interactions on 6p21.3 and interactions among SNPs on genes previously reported to be related with RA and other autoimmune diseases. For example, *RUNX1 *has been reported to be associated with systemic lupus erythematosus and psoriasis (two autoimmune diseases) [8,9] while associations of region 6q23 and *MICA *with RA has been reported by Thomson et al. [10] and Martinez et al. [11], respectively. Detecting genes and environmental factors interacting to increase the susceptibility to disease risk is a very challenging task for many reasons, particularly for the large size of the data and presence of confounding factors such as LD, presence of phenocopies, locus heterogeneity, and population stratification. Information theoretic methods have high power in detecting gene × gene interactions and have the advantage of being simpler and computationally faster; *KWII*-based interaction analysis has been employed in [3,4]. Also, our method can be used when the genetic and environmental variables have different numbers of classes or when the phenotype has more than two classes. Although we initially planned for a genome-wide analysis, given the large size of the data, we were able to execute only a few iterations using our computational resources. Therefore, we decided to follow a candidate-gene-based approach. We believe that with the help of additional hardware, it is possible to implement our search strategy in a distributed computing environment employing multiple processors and to explore many more interactions with moderate to low magnitudes that are potentially associated with RA.

## List of abbreviations used

GAW16: Genetic Analysis Workshop 16; *KWII*: *k*-way interaction information; LD: Linkage disequilibrium; RA: Rheumatoid arthritis; SNP: Single-nucleotide polymorphism

## Competing interests

The authors declare that they have no competing interests.

## Authors' contributions

PC developed the computational methods and carried out the statistical genetics analysis. AZ was involved in the development of the computational analysis. LS participated in the statistical genetics analysis and interpretations. MR conceived the study and was involved all aspects of design and coordination.
